# Early life programming of pain: focus on neuroimmune to endocrine communication

**DOI:** 10.1186/s12967-016-0879-8

**Published:** 2016-05-06

**Authors:** I. Zouikr, M. D. Bartholomeusz, D. M. Hodgson

**Affiliations:** Laboratory of Neuroimmunology, School of Psychology, The University of Newcastle, Newcastle, NSW Australia; Laboratory for Molecular Mechanisms of Thalamus Development, RIKEN BSI East Building 4F 409, 2-1 Hirosawa, Wako, Saitama 351-0198 Japan

**Keywords:** Pain, Psychoneuroimmunology, Neuroendocrinology, HPA axis, LPS

## Abstract

Chronic pain constitutes a challenge for the scientific community and a significant economic and social cost for modern societies. Given the failure of current drugs to effectively treat chronic pain, which are based on suppressing aberrant neuronal excitability, we propose in this review an integrated approach that views pain not solely originating from neuronal activation but also the result of a complex interaction between the nervous, immune, and endocrine systems. Pain assessment must also extend beyond measures of behavioural responses to noxious stimuli to a more developmentally informed assessment given the significant plasticity of the nociceptive system during the neonatal period. Finally integrating the concept of perinatal programming into the pain management field is a necessary step to develop and target interventions to reduce the suffering associated with chronic pain. We present clinical and animal findings from our laboratory (and others) demonstrating the importance of the microbial and relational environment in programming pain responsiveness later in life via action on hypothalamo-pituitary adrenal (HPA) axis activity, peripheral and central immune system, spinal and supraspinal mechanisms, and the autonomic nervous system.

## Background

Physiological pain plays an essential and primordial function in our survival. It signals potential damage to the body and produces a wide range of sophisticated actions to prevent this damage. The perception of noxious stimuli is typically initiated by the activation of peripheral receptors (i.e. nociceptors) which signal the spinal dorsal horn and supraspinal neurons of any potentially tissue-damaging stimuli. In pathological conditions, however, failure to sense pain such as in the case of congenital insensitivity often lead to self mutilation, bone fractures, joint deformities, amputations, and even early death, underscoring the importance of the physiological, protective role of nociceptive pain [1]. Chronic pain, the unbearable pain that persists long after an injury has healed, can drive the individual to immobility, psychological distress, depression, and disruption of family relationships, and potentially loss of employment. Chronic pain is common in developed countries. Prevalence rates of 16–22 % have been reported from epidemiological surveys [2–4]. One recent study lists chronic back pain (along with depression) in the top 10 causes of morbidity in over 180 countries [5]. Chronic pain incurs a multi-billion dollar cost to most developed western countries [6, 7]. Despite mounting evidence regarding the complexity of the nature of chronic pain there remains a general resistance [8] to the understanding that the nature of chronic pain is not primarily a matter of nociception (i.e. a noxious stimulus generating neural impulse), despite ample evidence of the multidimensional nature of chronic pain [9].

Pharmaceutical treatments, still our primary treatment modality, have a 25–30 % placebo rate, making therapeutic benefits difficult to establish. Some of the drugs such as the use of opioids (i.e. morphine), which can act on opioid-receptor-expressing neurons in brain areas that control pain (i.e. PAG) are effective but have a very narrow therapeutic window and in most of the time the dose needs to be limited to avoid side effects or analgesic tolerance which occurs upon repeated administration of opioids requiring increased dosage to maintain satisfactory level of pain control. Why in the 21st century is pain still a stubborn condition? What are the limitations of the current therapeutic approaches? Are we understanding and managing pain in its full complexity?

While our understanding of the complex mechanisms involved in the development and maintenance of chronic pain has increased dramatically in recent decades, treatments in various modalities continue to be based on a reductionist approach that understanding the precise nociceptive mechanisms that cause pain, will result in more targeted and hence more effective treatments. Given the myriad of neuroimmunoendocrine links now understood to modulate chronic pain, focusing on targeted specific mechanisms may be missing the bigger picture. Furthermore, given that pain has also a cognitive, aversive component there is a need to target the cognitive/emotional aspect of pain not only the sensory discriminative component.

From Galen to the gate-control theory developed by Melzack and Wall, pain has been viewed either as a separate, independent sensation, an emotion, or a result from activation of nociceptors, spinal and supraspinal pathways. This review proposes a shift in conceptualising the problem of chronic pain away from a dichotomous standpoint to an integrative view which captures both the sensory and the emotional-cognitive in a framework that emphasises the neuroendocrine to immune communication pathways and the relevance of early premorbid events as predictors.

Specifically we focus on the early life environment and its ability to program the organism’s physiological, affective and behavioural responses in later life [10–14]. Almost invariably, randomised control trials (RCTs) commence their assessment of pain by assessing the baseline values from the time the pain started. There is an implicit assumption, that prior to the onset of pain, “patients” are physiologically naïve and functionally equivalent—though there is some acknowledgement that their psychosocial history may differ. The thesis proposed here is that a neurodevelopmental understanding is required to account for the individual variability in response to the initiating noxious insult/stimulus as well as to subsequent treatments. We argue that not taking account of the premorbid physiological milieu may be limiting efforts to develop more tailored and more effective treatment interventions. In short, chronic pain patients may be differentially predisposed to recovering (or not recovering) from a physical injury, infection or trauma because of their premorbid psychoneuroimmunological status.

## The traditional theory of pain

### A brief historical overview

Aristotle (384–322 BC) considered pain to be an emotion. Later the Greek physician Galen (AD 130–201) using the experimental scientific method disagreed with Aristotle’s view and considered pain to be a sensation and the brain an organ for feeling. Avicenna (AD 980–1037) a renowned Persian philosopher, who is considered the father of modern medicine, argued that pain can be dissociated from touch. During the mid-1900s several studies performed by imminent Scottish and English neurologists demonstrated that hemi-section of the spinal cord resulted in a loss of pain sensation. The work by Blix and Goldcheider in 1884 and of Von Frey in 1897 demonstrated that different sensory experiences (i.e. pressure, cold, heat, pain) evoke the activation of different nerve endings. The terms noxious and nociception appears with Sherrington in 1906 who considered that noxious or painful events result in the activation of sense organs and this in turn produces pain [15]. In the 1930s, several studies by Bishop and Gasser demonstrated that noxious sensations are conveyed by two types of nerve fibers, namely Aδ and C [16]. It was with the development of the gate-control theory by Ronald Melzack and Patrick Wall in 1965 that the representation of pain as a unidirectional pathway from the site of injury to the sensation of pain was challenged. Instead pain was viewed as a result of supraspinal (brain) activity [17]. The development of new imaging techniques such as positron emission tomography (PET) and functional magnetic resonance imaging (fMRI) have revealed that pain results from activation of a number of brain regions such as the amygdala, insula or the anterior cingulate cortex.

Taken together, this brief historical outline illustrates the classical view that pain is an independent sensation, an emotion, a product of activation of nociceptors or spinal and supraspinal pathways. More recent conceptualisations of pain now view pain as a result of the complex interaction between the immune, nervous (both CNS and autonomic nervous system), and endocrine systems. Furthermore, a number of studies including findings from our laboratory clearly demonstrated that exposure to early life insults such as hindpaw inflammation, psychological/emotional or physical stress, as well as viral/bacterial infection can all lead to altered future pain responses [10, 18–24].

### Overview of chronic pain

Chronic pain described as a continuing (unremitting) presence for periods longer than 3–6 months, has been defined as: “an unpleasant sensory and emotional experience associated with actual or potential tissue damage or described in terms of such damage” by the International Association of for the Study of Pain (IASP).

Our current understanding of chronic pain is that it falls into one of 4 categories—nociceptive, neuropathic, psychogenic or a combination of these. Nociceptive pain arises from tissue injury, which activates nociceptors (pain receptors) in the periphery that send signals to the central nervous system (CNS). Nociceptive pain can be inflammatory or musculoskeletal in origin. Neuropathic pain, arises from damage to nerves that results in an altered quality (e.g. sharp, burning) and intensity of pain signals. However in clinical practice, these divisions are not easily discriminated from each other and often overlap. Nevertheless, the diagnostic divisions assist with directing therapeutic options.

Indicative of the complexity of chronic pain is the phenomenon of sensitization. There are two types of sensitization. The first type refers to peripheral sensitization which occurs in response to the release of a plethora of inflammatory molecules such as histamine, prostaglandins, pro-inflammatory cytokines which sensitizes (or increase the excitability) of nociceptors by creating an “inflammatory soup” environment which enhances pain sensitivity by reducing the threshold of nociceptors activation. The second type is central sensitization which refers to exacerbation of pain sensation and can be caused by hyperexcitability of spinal dorsal horn neurons or a decrease in inhibition of spinal dorsal horn neurons. The two phenomena of hyperalgesia (an exaggerated response to a normally painful stimulus) and allodynia (an exaggerated response to a normally non-painful stimulus) are clinical markers used to detect the presence of sensitization. There is now accumulating evidence that chronic pain sensitivity is elevated across different modes (e.g. pressure, thermal, pricking) within chronic pain populations. This has led to the use of quantitative sensory testing (QST), that attempts to measure pain related parameters such as pain thresholds, temporal summation and pain duration using repeated stimuli in multiple locations on the body and with multiple modalities (e.g. thermal, chemical, mechanical) [25]. So to illustrate, pain threshold can be evaluated by measuring the time taken for the person to report the sensation of pain once a graduated stimulus (e.g. temperature/pressure) is applied.

DNIC (one type of QST) is considered to be a marker of endogenous (possibly opioid) analgesia, and reflects the capacity of the CNS to modulate (down-regulate) nociceptive signals [25]. DNIC comprises the inhibition of a nociceptive test stimulus when a distant, usually contralateral noxious conditioning stimulus has been pre-emptively applied. In other words the first painful stimulus “dulls” the intensity of the second (“test”) stimulus. This reduction in intensity of the second test stimulus, is understood to be mediated by bi-directional opioid-sensitive neurons in the spinal-medullary-spinal pathway [26]. This capacity has been found to be reduced in a range of idiopathic pain related conditions such as tempero-mandibular disorders, fibromyalgia, tension head-ache, migraine and irritable bowel syndrome [26] as well as in a healthy non-patient population [27].

However attention is drawn to the predictive capacity of DNIC in post-surgical pain, which suggests that at least part of the pain response is determined by physiological factors that precede the onset of pain. The significance of this should not be overlooked, as this observation suggests that pre-existing vulnerability to cope with the surgical-related trauma. Consistent with this line of evidence, are recent observations from imaging studies that indicate that functional connectivity between the medial prefrontal cortex, (mPFC), and nucleus accumbens, (NAc) are also predictive of those who progress from sub-acute (<4 months) to chronic (>6 months) lower back pain [28, 29]. Again note that the altered connectivity precedes the onset of acute pain, again implicating premorbid factors involved in the transition from (sub) acute to chronic pain. There has been a long-standing debate whether the features of chronic pain populations (e.g. demographics, education, gender, depression, sleeplessness, not coping, fear of pain, catastrophization, job satisfaction, helplessness) explain the acute to chronic pain transition. While these factors collectively account for 25 % of pain CP intensity, and 33 % of total disability [30], it is notable that for chronic back pain, the most common site of pain [2, 3], there are cognitive-emotional cortical regions that account for 70–80 % of the variance of pain intensity and duration [31]. In other words, higher order processing (including of emotional elements) appears to be more salient in the transition to and maintenance of chronic pain than is commonly appreciated. It is therefore of concern when treatment of chronic (low-back) pain is primarily focused on linking structural and mechanical causes (that predict only 17 % of the pain intensity variance and is unrelated to disability [31], to behavioural manifestations of the pain. This may be more easily remembered by the phrase—‘the gain in the pain happens mainly in the brain’.

### Pain pathways

**Fig. 1:**
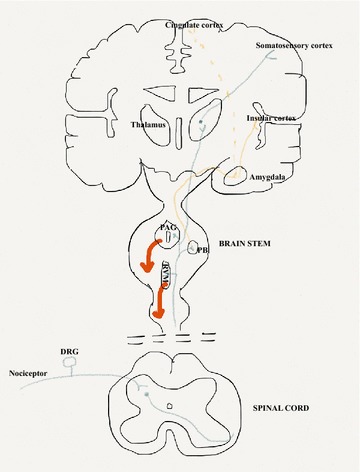
Anatomy of the pain pathways. Primary afferent nociceptors convey noxious information to neurons within the spinal dorsal horn of the spinal cord. These neurons project to the thalamus via the spinothalamic pathway and then to somatosensory cortex. This projection provides information about the location and nature of the stimulus (pathway in *blue*). The affective component is provided by projections to the cingulate and insular cortices via projections in the brainstem (parabrachial nucleus) and amygdala (pathway in *orange*). Descending inhibitory control pathways (*Red line*) originating in the PAG and RVM in the brainstem control the output of spinal nociceptive neurons in response to a noxious stimulus. *PAG* periaqueductal* grey*, *PB* parabrachial nucleus, *RVM* rostroventral medulla, *DRG* dorsal root ganglia

The conventional, traditional anatomical circuit of pain starts with the activation of nociceptors by multimodal noxious stimuli (i.e. thermal, physical, or chemical). There are two major classes of nociceptors. The first class includes thinly myelinated Aδ that are responsible for detecting well localized sharp, fast pain (speed of conduction: 5–30 m/s). The second category or C-fibers (unmyelinated; velocity less than 1 m/s) and belong to primary sensory neurons whose cell bodies are present in the dorsal root ganglia for the body or the trigeminal ganglion when innervating the face. Most Aδ fibres respond to heat as well as mechanical stimuli, whereas most C fibres are polymodal and respond to noxious thermal, mechanical, and chemical stimuli. Moreover, there are two main groups of C fibres: those that express the P2X3 purine receptor, the IB4-lectin-binding site and receptors for Glial Cell Derived Neurotrophic Factor (GDNF). This first group of fibres project exclusively to lamina II. The second group expresses peptides such as substance P and calcitonin gene related peptide (CGRP), but also expresses the receptor for nerve growth factor (NGF), neurotrophic tyrosine kinase receptor type 1 (TrkA). This group projects mainly to Lamina I. When activated, these peptidergic C fibres induce plasma extravasation and vasodilatation [30]. Many of the neurons in the marginal layer (Lamina I) respond exclusively to noxious stimuli and are called “nociceptive-specific neurons”, whereas some neurons respond to both noxious and non-noxious stimuli and are called “wide dynamic range” neurons. The substantia gelatinosa or Lamina II is made up exclusively of excitatory (glutamate) and inhibitory (gamma aminobutyric acid, GABA) interneurons. The nociceptive message, after being detected by peripheral nerves, is conveyed to neurons in the spinal cord that cross the spinothalamic tract (see Fig. 1) and make synapses in the thalamus. Once the message arrives at the somatosensory cortex, the conscious perception of pain occurs. The sensori-discriminative component of pain is integrated in the somatosensory cortex, whereas the emotional-affective component is coded by limbic structures such as the anterior cingulate cortex, amygdala, and insular cortex [31]. Other ascending pathways exist for the transmission of pain such as the one that initiates in the spinal cord and projects to the parabrachial nucleus, and then to the hypothalamus and amygdala; or the spinoreticular pathway that crosses the reticular formation in the medulla level, then thalamus and finally reaches the postcentral gyrus. In addition to these ascending pathways, the control of pain involves descending pathways also known as inhibitory descending control systems.

Figure 1 illustrates the complexity of the processing, as indicated by the number of tracts and regions involved. It would seem evident that there is no single anatomical region in the CNS that registers and processes nociception. This complexity would suggest processing far beyond that of mere auditing pain intensity and location. Also the involvement of the hypothalamus, thalamus, LC and parabrachial regions raises the possibility that all 3 stress-related neuroendocrine pathways listed above, being activated by pain. Finally activation of the prefrontal cortex (medial and lateral), the cingulate gyrus and insula cortex raises the distinct likelihood that higher order cognitive and emotional elements are involved with the processing of nociceptive stimuli.

In addition, recent studies using magnetic resonance spectroscopy have shown that there are differences in the neural processing between experimental pain within control subjects, and chronic pain in clinical populations. Afferent nociceptive information (via the thalamus) which projects to the somatosensory, insular and anterior cingulate cortices is more prominent in experimental pain conditions; by contrast projection to the prefrontal cortex (possibly via spinohypothalamic, spinoparabrachial and spinoreticular pathways) is more evident in chronic pain conditions [32]. Of note is that the prefrontal cortex (and thalamus) is associated with significant loss of grey matter in chronic back-pain patients [33]. What appears to be increasingly recognized is the importance of higher order processing of the nociceptive signals [34–36]. Therefore it is reasonable to examine conditions that have a pervasive and enduring impact upon the same higher order processing regions, prior to the onset of chronic pain. Childhood psychosocial stressors have such an impact upon adult neuroendocrine functioning and have been associated with the presence of chronic pain [37–42].

## Why pain treatment is ineffective?

Sixty-plus years of intensive research in the pain field have led to the discovery of countless drugs including aspirin and pioneer findings such as the induction of analgesia by direct stimulating of the periaqueductal grey (PAG) in rodents [43] and humans [44]. Opioids are effective in attenuating the noxious stimulus. These molecules (whether endogenous or exogenous) exert their action through activation of opioid receptors which are μ, δ (DOR/OPDR), κ (KOR/OPRK1), and opioid-like receptors (ORL1/OPRL) [45] that are expressed peripherally by leukocytes or by primary sensory neurons but also can be expressed centrally in key nuclei involved in the descending pain inhibitory pathways such as PAG and rostroventral medulla (RVM). However the use of opioids in modulating pain has a downside, they can be addictive and enhance alcohol consumption. They can also induce opioid tolerance where repeated exposure to opioids decreases drug efficacy over time. The use of slow-release drug patches, local anaesthetics, or spinal epidurals, which can act either by blocking the transmission of noxious input to the brain or by attenuating the excitability of spinal and supraspinal neurons have brought relief to some patients but not all. Surprisingly drugs that were traditionally prescribed to control depression were shown to be effective in treating chronic pain and the same discovery applied for drugs that were originally designed to treat epilepsy and were found to relieve severe chronic pain. These drugs shifted the focus onto the brain. However despite the effectiveness of these drugs, treating chronic pain is still challenging for clinicians. We argue that one of the reasons resulting from this challenge is the complexity of the interaction between multiple physiological systems that modulate pain as well as the role of early life events in programming pain responses later in life. Early life exposure to adverse events whether it is of psychological, physiological, bacterial or viral nature has been shown to exert programming effects on the hypothalamus–pituitary–adrenal axis [10, 46–48], a key system that modulates the stress response to physiological or psychological stressor. Given that pain is an aversive, stressful experience, these early adverse events are also capable of altering future pain responses through action on the endocrine system. Below we present clinical and animal evidence that demonstrate the impact of early life insult on neuroendocrine and pain responses.

## Perinatal exposure to adverse events alter the hypothalamus–pituitary–adrenal (HPA) axis later in life

The experience of stress, from an evolutionary perspective, plays a pivotal role in promoting survival of the organism. Perceiving an environmental stimulus as threatening and learning to avoid it is essential for survival. The HPA axis is the major neuroendocrine mediator of the stress response and is known to be susceptible to perinatal programming [49, 50]. The cascade of signalization in the HPA axis in response to stress begins with activation of a group of neurosecretory neurons in the paraventricular nucleus of the hypothalamus (PVN). These neurons secrete corticotropin-releasing hormone (CRH) and to a lesser extent arginine vasopressin (AVP) into the portal vessel system to reach the anterior pituitary where the synthesis of pro-opiomelanocortin (POMC) takes place and then processed to adrenocorticotropic hormone (ACTH) [51]. Subsequent increase in circulating ACTH then drives the synthesis of cortisol (in humans) and corticosterone (in rodents). Attenuation of the magnitude of the HPA stress response occurs through negative feedback of glucocorticoids on glucocorticoid (GR) and mineralocorticoid (MR) receptors expressed in neurons of the hippocampus, hypothalamus, and pituitary. This results in a negative feedback loop [52, 53] and maintains homeostasis. The HPA axis is therefore designed to provide a quick and appropriate response to stress by stimulating a relatively brief influx of glucocorticoids into the body. Moreover, this responsivity is determined, in part, by the relative affinity of glucocorticoids for MRs and GRs [54]. The HPA axis goes through a period of significant change and development during the perinatal period and exposure to adverse events during this period is known to produce long-term alterations in neuroendocrine function and behavior.

### Prenatal studies

#### Human studies

A growing body of clinical literature has demonstrated that exposure to stress during the perinatal period is linked to adverse health outcomes later in life [55–58] including susceptibility to developing psychiatric disorders and cognitive impairments. This is of particular relevance given that in modern society we are increasingly exposed to pollutants and stress, and thus it is proposed that the propensity for dysregulation of immune and metabolic homeostasis is rising.

The intrauterine events can have significant lasting or even lifelong repercussions such as cardiovascular disease or diabetes type 2 [59]. This delayed insult to the organism’s integrity is referred to as programming and is associated with sensitive periods during gestation. Various components of the stress related system develop at different rates. For instance, ACTH has been detected as early as 7 weeks, while corticotropin-releasing hormone CRH is produced by the placenta and hypothalamus and appears at 12–13 weeks [60]. The significance of these developmental stages for maternal stress is still unknown, however it is clear that levels of maternal glucocorticoids above that required for normal maturation [61] can have a negative “programming” impact on the foetus [53, 62]. These effects include growth retardation, HPA dysregulation, behavioural alterations and glucose intolerance in the offspring. [63]. Exposure to stress during pregnancy has also been linked to altered endocrine responses later in life. Maternal anxiety, depression, or stress have been associated with increased basal responsivity of the HPA axis in the offspring of children at 6 months [64], 5 years [65], and 10 years of age [58]. Pregnant mothers subjected to psychosocial stress showed alterations in HPA axis activity as indicated by high level of ACTH and decreased serum cortisol level when compared to a prenatally non-stressed group [66]. It has also been noted that social stress and lack of support during pregnancy, can increase IL-6 levels, increase CRH levels and decrease IL-10 levels within the maternal circulation [67].

#### Animal studies

The HPA axis goes through a period of increased plasticity during the perinatal period. Whilst GR mRNA in the hypothalamus can be detected as early as gestational day (GD) 16 [68], MR mRNA is not detected until PND 2 [69]. Thus, exposure to stressors during this sensitive period of development can have long-term consequences on the HPA axis. Physiological levels of glucocorticoids play a crucial role in brain development. They are involved in axonal and dendritic remodeling, and can affect cell survival [70]. However, sustained increases or removal of glucocorticoids during development can permanently alter brain structure and function [49, 71]. For instance, administration of the glucocorticoid synthetic Dexamethasone in pregnant rats contributed to delay in the maturation of neurons, myelination, glia, and vasculature in the offspring, leading to altered neuronal cytoarchitecture, altered synaptogenesis, and attenuated levels of neurogenesis [72]. Furthermore, adult rats prenatally exposed to glucocorticoids displayed increased CRH levels in the central nucleus of the amygdala [73], a key region in the regulation of the emotional component of pain [74]. Exposure to a novel environment combined with a saline injection (mild stress) from GD14 to GD21 is also associated with elevated plasma levels of ACTH and corticosterone in adulthood. Moreover when offspring from prenatally stressed rats were exposed to an acute foot shock, they exhibited a greater amount of shock-induced freezing time than the control group [75].

### Postnatal studies

#### Human studies

The hippocampus is a key regulator of the stress response having inputs into a variety of key loci in the brain that regulate behavioural and endocrine responses to stress [76, 77]. The hippocampus represents a major regulator of HPA axis activity. Activation of glucocorticoid (GR) and mineralocorticoid (MR) receptors by circulating glucocorticoids (e.g. corticosterone) provides a negative feedback, which serves a key role in maintaining normal functioning of HPA axis. A number of studies have shown that exposure to adverse life events such as poverty [78], abuse [79], or orphanage rearing [80] can lead to children with increased stress reactivity and dysregulated HPA function in adulthood. For instance, in an aged human population, long-term exposure to endogenous levels of glucocorticoids resulted in memory impairments and a significant decrease (14 %) in hippocampal volume [81]. Low birth weight combined with low maternal care contributed to smaller hippocampal volume in adulthood, leading to increased susceptibility to stress-related disorders in later life [82, 83]. Maternal emotional well-being can also affect the development of the hippocampus in children. Magnetic Resonance Imaging (MRI) studies revealed that children from mothers who experienced high level of anxiety during the last trimester of pregnancy exhibit slower growth of both hemispheres of the hippocampus over the first 6 months of postnatal life [77]. Decreased hippocampal volume has also been associated with being sexually abused at 3–5 and 11–13 years of age [84]. Parent–child interactions and the emotional state of the pregnant mother are also critical factors in determining HPA axis responsivity of the child later in life. There is increasing evidence that children from depressed mothers are at higher risk of increased HPA axis activity [78].

#### Animal studies

In rodents, the first two postnatal weeks constitute an important time window for HPA axis ontogeny whereby the HPA axis goes through a “stress hyporesponsive period” (SHRP) from PND 4 to PND 14 during which adrenal sensitivity to stressors is diminished and corticosterone concentrations are maintained at low levels [85]. Furthermore, the maturation of hypothalamic neurocircuits is not completed at birth and undergoes fine-tuning until the third postnatal week. Whilst the neuronal cell population in the hypothalamus is determined prior to birth, the formation of functional neuronal networks via axonal projections and synaptic connections occurs later during the postnatal period [86]. Adult rats challenged with LPS as neonates displayed increased CRH mRNA in the PVN, indicating decreased levels of negative feed-back and hence increased HPA axis activity [47]. Maternal care constitutes a major determinant of an individual’s development, shaping the phenotype by affecting brain structure and function and ultimately determining personality, physiology and behaviours. Maternal separation, an animal model of human child maltreatment, has been linked to increased density in CRH binding sites in the hippocampus, prefrontal cortex, amygdala, and hypothalamus, as measured post-infancy [48]. Daily handling from PND 2 to PND 8 resulted in down-regulation of hypothalamic CRH mRNA levels in PND 23 rats [46]. Our laboratory was recently able to demonstrate that exposure to the bacterial mimetic LPS at PNDs 3 and 5 can also alter HPA axis in a developmentally regulated way as indicated by increased circulating corticosterone at PNDs 7 and 22 but not 13 and decreased GR mRNA and increased MR mRNA in the hypothalamus of PND 22 rats following formalin injection [10]. Similar findings were reported by other researchers whereby LPS exposure during PNDs 3 and 5 resulted in decreased GR binding and density in the hippocampus, hypothalamus, and frontal cortex of adult rats following adrenalectomy [47]. Moreover, adult rats challenged with LPS as neonates displayed increased CRH mRNA in the PVN, indicating decreased levels of negative feed-back and hence increased HPA axis activity [47].

Taken together, animal and human studies suggest that exposure to stress during the perinatal period where key brain areas are still developing at the time of stress exposure, can have long-term consequences on HPA axis activity, leading to exaggerated responses to a stressful event encountered later in life. The effects on HPA axis depend on the nature of the stressor as well as the timing when it occurs (i.e. prenatal vs. postnatal).

## Plasticity of the nociceptive system during the neonatal period

Nociceptive neural circuitries undergo significant changes during the early neonatal period, and therefore remain vulnerable to the effects of early insults. For instance, substance P, a specific marker of peptidergic C fibres, is present at birth suggesting the development of chemical properties of these fibres at an early stage. The peptidergic C fibers are a population of C fibers that release neuropeptides such as substance P and calcitonin-gene-related-peptide (CGRP) as well as the Trk A neurotrophin which binds to nerve growth factor (NGF) [31]. In the dorsal horn, substance P fibres are concentrated in Lamina I on day 1; in the next few days, the density in Lamina II is increased and by the end of the first week of development, the staining of the fibers is comparable to the one observed in adults [87]. Moreover, the non-peptidergic C fibres that express the IB4 isolectin cannot be detected in the synapse until PND 5 despite their presence in dorsal root ganglion from embryonic day 18. This delay suggests that these fibres form synapses later than peptidergic C-fibres. Finally, both neonate A-fibres and C fibres express µ-opioid receptors, although this expression becomes restricted to C fibres by PND 21 [88].

There is a functional maturation of spinal dorsal horn which is also developmentally regulated. Dorsal horn cells in deep laminae do not respond to C fibre input until PNDs 7–8. Similarly, specific C-fibre-evoked reflexes produced by mustard oil (a chemical skin irritant) do not occur until rat pups reach day 10–11 [89]. The activation of C fibres is unable to evoke spike activity in the spinal cord before the second postnatal week. Aδ fibres are completely unmyelinated until the pups reach 2–3 weeks [89]. In the mature state, Aβ fibres which convey non-noxious stimuli project into Laminae III to V [90], while nociceptive fibres terminate in the superficial laminae (Laminae I and II). However, in the immature state large myelinated fibres, which normally integrate non-painful stimuli such as touch, are found to project to Laminae I to V including Laminae II. In other words, fibres that normally convey non-noxious stimuli can integrate noxious information as well. At the supraspinal level, the inhibitory descending control system is not apparent until the second postnatal week. Interestingly, electrical stimulation of the PAG does not produce analgesia until at least PND 10 [91]. Of particular interest, a recent study showed that there is a developmental postnatal shift in the brainstem rostroventral medulla (RVM) control of spinal dorsal horn neuronal circuits from A fibre facilitation activity in young animals to inhibition of nociceptive C fibre input in the adult animal [92].

Taken together, these data demonstrate that the nociceptive system during early life presents an important “window of vulnerability” during which an early insult such as inflammation, bacterial infection or a polymodal stimulus (mechanical, chemical or thermal), may result in the disruption of the neuronal and chemical circuitry modulating pain, leading to altered behavioural and neuronal responses to noxious stimuli later in life.

## Neonatal noxious stimuli alter future pain responses

### Human studies

In contrast to the traditional view that argues neonates are unable to feel pain, it is now well acknowledged that neonates are neuroanatomically and functionally able to discriminate between noxious and innocuous stimuli as indicated by scalp electroencephalography and spectroscopy studies [93, 94]. Preterm infants (<37 weeks of gestation) are born during a critical period when the brain is still undergoing significant plasticity and development. Preterm infants admitted to the neonatal intensive care unit (NICU) are routinely exposed to stressful and invasive procedures (i.e., heel lance, chest tube insertion, tape removal, and nasogastric tube insertion) to maintain their survival [95]. These infants exhibit significant alterations in sensory and pain processing later in adulthood [96, 97]. For instance infants subjected to repeated heel lance develop hyperalgesia compared to non-injured controls [98]. A number of studies have demonstrated that exposure to such adverse events can contribute to altered brain neurocircuitry and processing, leading to poorer outcomes at school age [99–101]. For instance infants, children, and preadolescents born preterm were found to exhibit altered development in the cerebellum and reduced cerebellar volume [102]; reduced white matter integrity [103]; as well as significant deficits in executive function and memory [104].

### Animal studies

Animal studies are in accordance with the above-mentioned clinical findings whereby full thickness skin removal (wound of 2 mm in diameter) in rats performed at birth results in hyperinnervation of the wounded area that persist long after the wound has healed (i.e. up to 12 weeks). This hyperinnervation involved both Aδ and C-fibers and paralleled a decrease in the threshold to mechanical stimuli applied to the injured area [105]. Neonatal hindpaw injury produces hyperalgesia in adulthood [20] and intrathecal administration of minocycline, which inhibits microglial activity, at the time of adult insult prevent this hyperalgesia [106]. Finally, adult rats treated with the persistent inflammatory-inducing agent, Compound Freund Adjuvant (CFA), at PND 1 displayed increased density of dorsal horn nociceptive primary afferents [107]. And electrophysiological recording of dorsal horn neuron from adult rats treated with CFA as neonates revealed enhanced evoked responses to brush and noxious pinch compared to untreated rats [107].

The mechanisms underlying the hyperalgesia following early life adverse events are numerous. The hyperalgesia can be mediated by peripheral nerve sprouting such as the innervation of nociceptors is increased as previously confirmed [89]. Also, the ensuing inflammation may contribute to enhanced dorsal horn central sensitization as Ruda et al. previously reported [107]. Furthermore, the hyperalgesia can result from altered descending inhibitory control systems and decreased opioidergic drive in the PAG. Finally and of particular importance to this review the altered pain responses can result from altered neuroimmune responses as described below.

## Neuroimmune interface in pain

Traditionally implicated in defending the organism against microbial pathogens or “non-self”, the immune system is now well appreciated to play a critical role in pain modulation [108–110]. Following injury, immune cells release proinflammatory cytokines and other inflammatory molecules such as histamine, prostaglandins, eicosanoids and cytokines that sensitize nociceptors. For instance, nociceptors are known to be interleukin (IL)-1-β sensors and IL-1-β can directly activate nociceptors to generate action potentials and induce hyperalgesia [111]. Moreover, intraplantar injection of IL-1-β increase the discharge of spinal dorsal horn neurons to thermal and mechanical stimuli [112]. The contribution to pain hypersensitivity can be mediated by inflammatory mediators released by immune cells but also by immune cells infiltration per se. The release of cytokines contributes to the recruitment of granulocytes, monocytes and mast cells at the site of inflammation. An increasing body of evidence suggests that infiltration by macrophages and T-cells in the dorsal root ganglia following nerve injury [113, 114] as well as activation of microglial cells in the spinal cord that coincides with enhanced neuropathic pain [115]. Hyperalgesia following nerve injury is substantially reduced in mice lacking T cells [116–118] and following the inhibition of microglia by minocycline [119]. Infiltration of immune cells at the peripheral level is also important in contributing to pain modulation. Following injury, mast cells are first to infiltrate the site of inflammation and they can degranulate within minutes of inflammation leading to the release of histamine, prostaglandin, bradykinin and other inflammatory markers that participate in the vasodilation and nociceptors sensitization [120]. Interestingly, mast cell degranulation requires direct contact with nociceptors via molecule of adhesion (N-cadherin) [121]. In our laboratory, we have demonstrated that neonatal exposure to the bacterial mimetic lipopolysaccharide (LPS) produced hyperalgesia in preadolescent rats that coincided with enhanced mast cell degranulation [23]. In a recent clinical study, Di Nardo et al. showed that children suffering from Irritable Bowel Syndrome (IBS) exhibit an increased number of mast cells in the proximity to nerves in the ileal and colonic mucosa [122]. More importantly, the intensity of abdominal pain reported by these children was significantly correlated to the close proximity of mast cells to nerve endings in the gut wall [122].

Other events that can contribute to the induction of the immune system are exposure to viruses, lipopolysaccharide (LPS), cell wall of gram-negative bacteria, or fungal infections during both the neonatal and adult life. When exposed to such components, immune cells including monocytes, lymphocytes, leukocytes, macrophages, T cells and mast cells not only signal other components of the immune system (i.e. complement) but also the brain [123] to control the inflammatory response and to maintain it at homeostatic levels. This is made possible by the release of a plethora of pro-inflammatory cytokines including IL-1-β [124], IL-6 [125], IL-8 [126], IL-12 [127], interferon-α (IFN-α) and -δ (IFN-δ) [128] as well as tumor necrosis factor (TNF)-α [129, 130]. Since cytokines are large molecules that are unlikely to cross the blood brain barrier (BBB) [131], it has been postulated that they can act on the brain through different pathways. For instance, via the synthesis of prostaglandin E2 by brain microvessel endothelial cells [132]. Another possibility is through vagal afferences as subdiaphragmatic lesions of the visceral vagal afferents terminating in the nucleus tractus solitaris abolished the LPS or IL-1β-induced hyperalgesia [133]. Subdiaphragmatic lesions of the vagus nerve also abolishes expression of Fos-protein in the secondary projections of this nerve such as the paraventricular nucleus of the hypothalamus (PVN), central nucleus of the amygdala (CeA), and the bed nucleus of the stria terminalis following IP injection of LPS [134]. This indicates that systemic LPS requires an intact functional vagus nerve to induce neuronal activation of brain regions involved in neuroendocrine function such as the PVN. It has also been suggested that cytokines can reach the brain via areas devoid of BBB such as the organum vasculosum lamina terminalis (OVLT) [135] or the macrophage-like cells commonly known as circumventricular organs [136]. The brain production of IL-1β following peripheral inflammatory stimuli such as in the case of LPS administration can be first restricted to choroid plexus and circumventricular organs, and then to the brain side of the BBB by slow diffusion (via volume transmission) [137]. Finally cytokines can reach the brain through a saturable transport system, which although has been demonstrated to exist in mice but not rats [138, 139], its physiological relevance remains unclear.

All of these neuroimmune pathways ultimately lead to the production of cytokines by brain microglia and induce a set of physiological responses commonly known as “sickness behavior”, which include fever, malaise, lethargy, decreased appetite and libido [123, 140]. The sickness response is an adaptive mechanism assisting in the recovery process from acute viral/bacterial infections. For instance fever, induced by the action of the pyrogenic IL-1 on anterior hypothalamus, elevates the body’s temperature to assist the immune system in fighting infections by blocking the growth of microorganisms [141]. Fatigue helps conserve energy so much needed in order to fight infection. However, in some circumstances, this process can go seriously wrong such as in the case of uncontrolled, exaggerated and prolonged inflammatory response in the CNS. This maladaptive response can be exemplified in clinical settings of post-infectious chronic fatigue syndrome [142], irritable bowel syndrome [143], or post-viral depression [144]. Importantly, pain facilitation or hyperalgesia is also considered to be an integral part of sickness behavior [145, 146]. Proinflammatory cytokines released by immune cells are known to induce hyperalgesia when administered at the peripheral or central level. Among the many types of pro-inflammatory cytokines, IL-1β is the most characterized potent mechanical and thermal hyperalgesic agent when injected into peripheral tissue or the CNS [112, 133, 147, 148]. For instance, intracerebroventricular (ICV) injection of the recombinant human IL-1β (rhIL-1β) in rats significantly decreased paw lick latency to thermal noxious stimulus [148]. This thermal hyperalgesia was abolished by ICV administration of the IL-1β antagonist IL-1ra [148]. In our laboratory, we have demonstrated increased formalin-induced behavioural responses in LPS-treated adult rats that paralleled enhanced circulating IL-1β 1-h following formalin injection [23]. Additionally, adult rats treated with LPS at PND 3 and 5 displayed increased IL-1β in the hippocampus 1 h following formalin injection, an effect that coincided the increased formalin-induced nociception observed in adult LPS-treated rats. The neurocircuitry underlying central action of cytokines is not only restricted to the brain but can also involve the spinal cord by increasing the excitability of spinal dorsal horn neurons [149, 150]. As discussed previously, activation of microglia at the spinal level plays a critical role in producing this cytokine-induced hyperalgesia [151].

## The impact of neonatal and adult LPS exposure on pain responses

Neonatal or adult exposure to the bacterial mimetic has been reported in both clinical and animal studies to produce increased pain sensitivity later in life. Boisse L et al. were the first to report that rats administered with LPS at PND 14 exhibited a significant decrease in withdrawal latency to thermal and mechanical stimuli in adulthood [152]. These adult rats displayed allodynia (a stimulus that is normally not painful is perceived as painful) and hyperalgesia (exaggerated responses to stimuli) in both tests of thermal (i.e. hot-plate test) and mechanical nociception (i.e. Von Frey test). Of particular interest, no significant differences were observed in nociceptive responses when a second LPS injection was administered in adulthood. This implies that a single LPS administration is enough to produce long-term alterations in nociceptive responses. Moreover, the thermal and mechanical hyperalgesia in neonatally LPS-treated rats was associated with upregulation of COX-2 protein level in the spinal cord [152], suggesting a potential role of prostaglandins in the hyperexcitability of spinal dorsal horn neurons in LPS-treated rats. We have replicated these findings such that rats exposed to LPS during PND 3 and 5 displayed increased formalin-induced nociceptive responses (i.e. flinching and licking) following formalin injection at PND 22 and PND 80–97 but not PND 7 or PND 13 [10, 23, 24]. This LPS-induced hyperalgesia was accompanied by peripheral and central neuroimmune and neuroendocrine changes as well as altered spinal and supraspinal neuronal responses [10, 23, 24]. More recently Hunter D and colleagues reconfirmed these findings showing that at 4 h and 8 h post LPS administration, PND 21 rats (PND0 as birth) displayed enhanced formalin-induced nociception during the late phase [153]. Adult LPS administration in rats is also reported to significantly decrease the tail-flick latency, which starts within few minutes of LPS injection and lasts for 60 min [154]. The LPS-induced hyperalgesia can be blocked following administration of IL1-ra, suggesting a critical role of IL-1 in inducing the LPS-induced hyperalgesia [155]. These animal studies are consistent with clinical findings showing that humans subjected to an IV injection of LPS exhibited enhanced flare, hyperalgesia, and allodynia in response to capsaicin administration [156] as well as lower pressure pain thresholds that correlated with enhanced circulating levels of IL-6 and TNF-α, an effect more pronounced in women [157]. This is consistent with a recent study, which demonstrated that IV injection of LPS produced a significant decrease in rectal and pressure pain thresholds [158]. This LPS-induced visceral hyperalgesia was accompanied by a significant increase in circulating pro-inflammatory cytokines as indicated by a peak of TNFα and IL6 1 and 2 h (respectively) following LPS administration [159].

Taken together, these animal and clinical findings suggest that far from the traditional view that posits that pain can solely be attributed to activation of neurons, the immune system via peripheral and central release of pro-inflammatory cytokines contribute significantly to pain modulation. Integrating the neuroimmune interface in pain modulation is thus a necessary step in order to find efficient treatments to treat chronic pain.

## Neuroendocrine-immune crosstalk: Focus on IL-1β and implications for pain

During the late 80s, a series of elegant studies mainly carried out by Sapolsky and Besedovsky group have demonstrated that components of the immune system, namely cytokines and in particular IL-1β can directly activate the HPA axis leading to the release of glucocorticoids [160, 161]. In a clinical study on post-mortem tissue, immunohistochemistry staining using antiserum directed against human IL-1β revealed that the human hypothalamus is densely innervated by IL-1β fibers [161]. The densest innervations were found in the periventricular region that controls the anterior pituitary. IL-1β-immunoreactive fibers were also found in the arcuate nuclei and parvocellular part of the paraventricular nucleus [161]. In rats, intravenous injection of IL-1β induced *cfos* expression in corticotropin-releasing hormone (CRH) containing neurons in the paraventricular nucleus of the hypothalamus (PVN) [162]. Systemic or intracerebroventricular injection of IL-1β also induced the release of circulating corticosterone and adrenocorticotropic hormone (ACTH) [163, 164]. Moreover, immunoneutralization of IL-1β or CRH blocked the stimulatory effect of IL-1β on ACTH [164]. This response is primarily mediated by the release of CRH from the PVN [164]. Additionally, IL-1β has been reported to act directly on the anterior pituitary cells to release ACTH [165].

Adrenocorticoids such as cortisol in humans and corticosterone in rats are known for their immunosuppressive function [166]. However their exact contribution to pain modulation is still not clear and literature regarding the role of glucocorticoids in modulating pain is at best conflicting. It is not surprising that glucocorticoids play an important role in pain modulation since pain is an aversive stressful event and hence capable of activating the HPA axis. Whilst several clinical studies have reported hypocortisolemia in patients with fibromyalgia and chronic low back pain [167–169], others have found that corticosterone reduces the inflammation associated with pain [170, 171]. In our laboratory, we demonstrated that neonatal LPS exposure produced a developmentally regulated activation of HPA axis activity following a noxious stimulus (i.e. formalin injection) as indicated by increased plasma levels of corticosterone in PND 13 and 22 but not PND 7 rats [10]. Preadolescent rats neonatally treated with LPS also exhibited decreased GR mRNA transcripts in the hypothalamus compared to rats that had saline during the neonatal period [10]. This is in agreement with previous studies indicating that noxious stimuli including formalin injection are capable of activating the HPA axis [172–174]. These findings indicate that the relationships between peripheral HPA axis activity and pain modulation is complex. Further studies investigating the exact contribution of the HPA axis in pain modulation are needed.

## Conclusion and final remarks

**Fig. 2:**
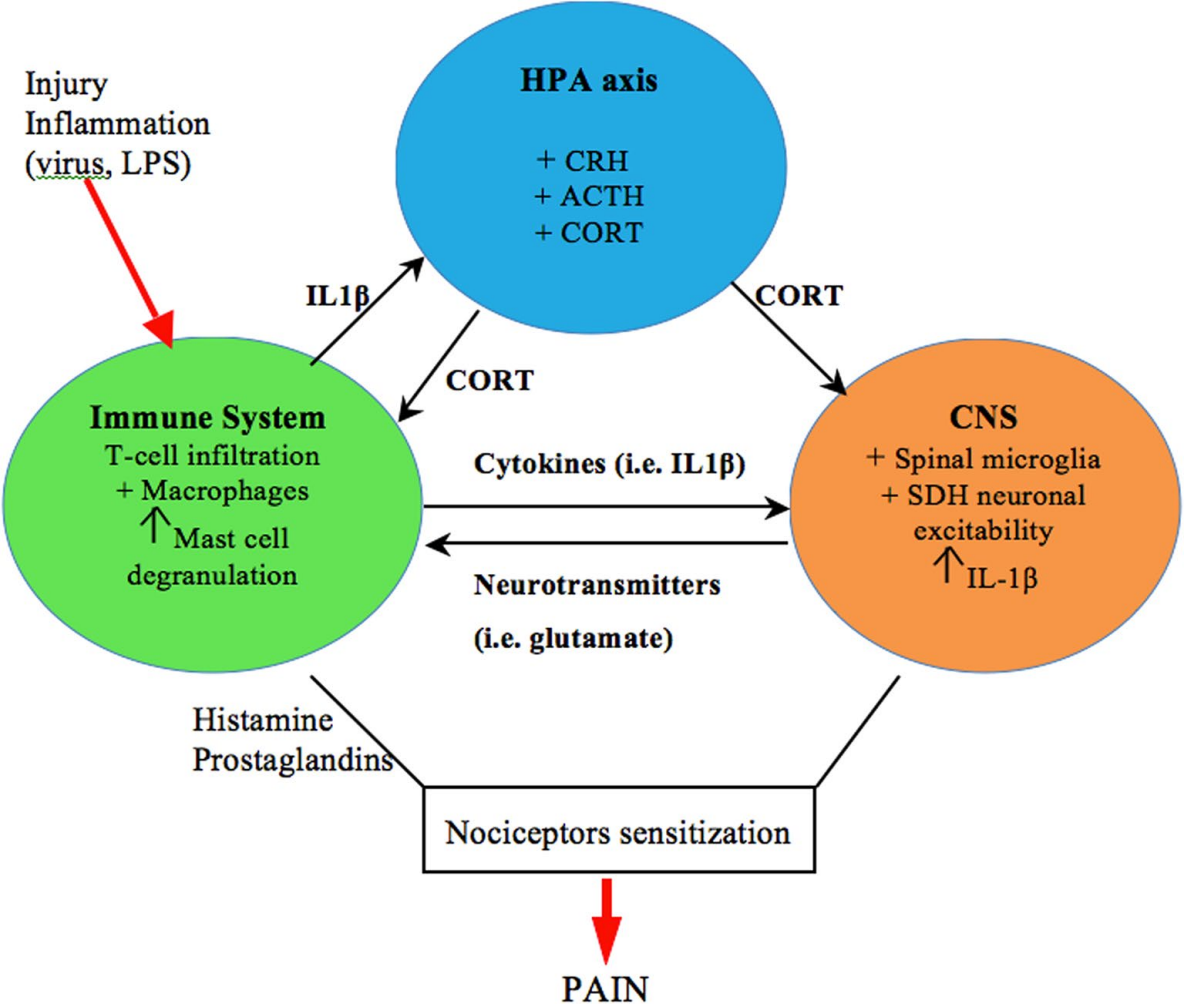
Neuroendocrine to immune communication in pain. Following a viral or bacterial infection, immune cells (i.e. T cells, macrophages, mast cells) are activated and infiltrate the site of inflammation. This results in the release of proinflammatory cytokines such as IL-1β. IL-1β then activates spinal microglia, produces the excitation of spinal dorsal horn (SDH) neurons and via activation of the HPA axis results in the release of corticosterone. Immune cells also release a plethora of inflammatory molecules including histamine and prostaglandins which results in the sensitization of nociceptors and increased pain sensitivity

As human beings, our organism is very complex. Far from the traditional view that posits that every physiological system is self-regulated, every physiological system including the immune, endocrine, and nervous system are interconnected via well-organized and harmonious regulatory mechanisms. In fact, dysfunction in one physiological system will lead to dysfunction of another. The immune system communicates with the endocrine and nervous systems mainly via cytokines which can act directly on the brain to produce local neuroinflammation but also exert a direct action on the HPA axis to produce the release of cortisol. Given that the immune, endocrine, and nervous systems undergo significant plasticity during the perinatal period, exposure to adverse events during this critical window of development is susceptible to disturb the normal developmental trajectory of these systems and consequently disturb the highly organized neuroendocrine to immune network leading to long-term consequences including altered pain responses. The challenge now facing the scientific community and clinicians is how to translate the well-established concept of psychoneuroimmunology and psychoneuroendocrinology as well as the importance of early life events in programming future pain responses into therapeutic approaches to treat chronic pain. Instead of the reductionist approach, which consists of targeting neurons only, we can also target components of the endocrine system such as corticosterone blockers or components of the immune system such as blocking the inflammatory process via suppressing the activity of pro-inflammatory cytokines or enhancing the activity of anti-inflammatory cytokines (Fig. 2).
